# Strengthening the evidence-policy interface for patient safety: enhancing global health through hospital partnerships

**DOI:** 10.1186/1744-8603-9-47

**Published:** 2013-10-16

**Authors:** Shamsuzzoha B Syed, Viva Dadwal, Julie Storr, Pamela Riley, Paul Rutter, Joyce D Hightower, Rachel Gooden, Edward Kelley, Didier Pittet

**Affiliations:** 1 African Partnerships for Patient Safety, Patient Safety Programme, World Health Organization, Geneva, Switzerland; 2 Infection Control Programme and WHO Collaborating Centre on Patient Safety, University of Geneva Hospitals and Faculty of Medicine, Geneva, Switzerland

**Keywords:** Patient safety, Global health, Policy-making, Evidence-based practice, Africa, Hospitals

## Abstract

Strengthening the evidence-policy interface is a well-recognized health system challenge in both the developed and developing world. Brokerage inherent in hospital-to-hospital partnerships can boost relationships between “evidence” and “policy” communities and move developing countries towards evidence based patient safety policy. In particular, we use the experience of a global hospital partnership programme focused on patient safety in the African Region to explore how hospital partnerships can be instrumental in advancing responsive decision-making, and the translation of patient safety evidence into health policy and planning. A co-developed approach to evidence-policy strengthening with seven components is described, with reflections from early implementation. This rapidly expanding field of enquiry is ripe for shared learning across continents, in keeping with the principles and spirit of health systems development in a globalized world.

## Introduction

Global health continues to redefine itself from the historical legacy of 'international health’ and 'humanitarian medicine’. A process of 'co-development’ and mutual learning between countries is now needed to strengthen health systems and improve health outcomes. Koplan sees this evolution as running “parallel to a shift in philosophy and attitude that emphasises the mutuality of real partnership, a pooling of experience and knowledge, and a two-way flow between developed and developing countries" [1]. Lord Nigel Crisp also eloquently highlights the utility of shared learning to secure global health improvements [2]. This concept of bidirectional learning between rich and poor countries is increasingly being explored in emerging literature [3-6].

Whether in Mombasa, Manchester, or Miami, effective health systems are critical to improving population health. As health systems increase in complexity, public demands for accountability and performance too have grown, positioning the evidence-policy interface at the core of health systems development. However, many evidence-based, cost-effective interventions fail to capture the attention of decision-makers who ostensibly operate on diverging sets of cultures, interests, and imperatives [7]. Despite the fact that it is widely recognized that a strong evidence-policy interface is required to improve population health, fundamental differences between 'evidence generators’ and policy-makers complicates the translation of knowledge and practice into policy [7,8]. Complex as it may be, this interface is a critical ingredient in effective health systems development and requires the engagement of practitioners, policy-makers, and politicians alike [9,10].

The divide between evidence and policy remains substantial in many low and middle-income countries [11]. Obstacles to an effective evidence-policy interface, particularly in low-income countries include researcher-policymaker mistrust; low value placed on research data by policy makers; lack of awareness of available evidence (particularly in-country research); and political instability affecting policy processes [7,12-14]. Evidence-policy frameworks can help recognize these multiple influencing factors, which span structures, processes and individuals [15,16]. These influencing factors can be further understood using stakeholder analyses that assess unique influences of particular individuals within each setting. Resultantly, the need for engagement with stakeholders increases [17], and in times where a wide array of stakeholders exists, more formal strategies may be required for knowledge translation [13].

One such strategy is the use of 'knowledge brokers’ – intermediary organisations or individuals who integrate best available evidence into policy-making processes [7,18]. As catalysts, knowledge brokers look for, and nurture relationships so as to promote linkage and exchange along mutually beneficial lines [7]. The World Health Organization’s (WHO) Evidence Informed Policy Network (EVIPNet) is one such example of a network, whose aim is to support systematic, multifaceted efforts to address the challenges in linking research to policy [15]. Regional networks and country teams (e.g., EVIPNet Africa) also play a critical role in emphasizing local ownership and contextualizing evidence to build sustainable capacity in geographic areas [19] through the production of key instruments (e.g., 'policy briefs’ and 'capacity building workshops’) [20,21].

### Patient safety in low-income settings

Patient safety is a core component of an effective health system. Yet it is also a young discipline, particularly in developing countries. Successfully identifying the ingredients needed to improve patient safety has proven elusive, even within high-income settings [22,23]. Traditional approaches to patient safety have focused primarily on research to demonstrate how a new practice leads to improved quality and patient safety. Much less attention has been paid on how to implement these practices [24]. The importance of considering improvement as a socio-behavioural process has also been emphasized, but remains an emerging field of enquiry [25].

As the global knowledge in this field expands, the implementation of evidence-based practices in patient safety require targeted strategies that can address the cultures and complexities inherent in crosscutting systems of care [5,24]. Given that much of the evidence base for patient safety research comes solely from the developed world [26], more work is needed to understand the scope of solutions for the rest of the world—particularly for resource-poor settings. In this regard, testing and developing standardized patient safety strategies, tools, and measures is a priority at every level—the global, regional, national, and institutional [26].

Patient safety is gaining importance in Africa. In order to keep the momentum, sensitizing opinion leaders about the importance of evidence-based strategies has become critical. In 2008, the WHO African Region articulated a commitment to translating patient safety evidence into effective policy [27]. African health care professionals are adding their voice to this call [28], elevating the need to implement strong mechanisms that can bring together end-users, practitioners, and senior leadership to enable change. Indeed, the involvement of clinicians in swaying opinion leaders and multidisciplinary teams about evidence-based strategies has helped increase the use of evidence in practice [29].

### Strengthening the evidence-policy interface: African Partnerships for Patient Safety

African Partnerships for Patient Safety (APPS) forms part of WHO’s commitment to improving patient safety across Africa. Unlike other patient safety initiatives to date, this programme seeks to catalyse change through the establishment of mutually beneficial hospital partnerships [30]. The three core objectives of APPS focus on: first, building strong patient safety partnerships between hospitals in Africa and other regions of the world; second, implementing patient safety improvements in each partnership hospital; and third, facilitating spread of these patient safety improvements across countries.

The programme was co-developed by six hospitals spanning Africa (Cameroon, Ethiopia, Malawi, Mali, Senegal, Uganda) and Europe (England and Switzerland) [31]. Hospital partners quickly realized that facilitating the spread of patient safety thinking in Africa is contingent on strengthening the linkages between patient safety evidence and national health policies. Today, these intercontinental partnerships provide a clear opportunity to develop mechanisms that can strengthen and inform the evidence-policy interface—in both developing and developed country settings.

Insights on how knowledge-brokering networks help strengthen the complex interface between evidence and policy are only recently emerging [32]. Even less is known about how to strengthen this interface within low-income countries in a sustainable and meaningful way. APPS prioritized strengthening the evidence-policy interface as a critical crosscutting issue from its inception, considering it central to national spread of patient safety approaches that improve health service delivery. Ten key evidence-policy factors informed APPS programming (Table 1).

**Table 1:** **Key evidence**-**policy factors in APPS programming**

	
1	Recognition of the complexity of policy making processes
2	Understanding multiple types of evidence utilized for policy
3	Importance of developmental contexts
4	Need for generating and packaging evidence to provide realistic policy options
5	Necessity for trust-nurturing platforms that include evidence generators & policy makers
6	Understanding diverse stakeholders involved at the interface
7	Importance of communication (verbal and written) and use of standardized tools
8	Influence of accountability factors in interface strengthening
9	Critical need for evaluation and continuous learning
10	Potential for working with current structures in Africa

The programme further translated these factors into a step-wise approach that assists hospital partnerships to strengthen the evidence-policy interface on patient safety [33]. This approach, with its seven components, was built into the programme’s design from inception, in recognition of the often excellent but isolated work of hospital partnerships in stimulating change. Including this aspect from the start sought to ensure that the rich experiences of hospital partnerships would remain supportive of in-country evidence-policy strengthening efforts. The approach takes into consideration substantial input from front-line workers, as well as partnership-focused organizations. To our knowledge, this is the first time such an approach has been developed for use in hospital partnerships. Each of the seven components of the approach are outlined below with some early reflections on their implementation.

First, integrate "evidence-policy" considerations into programme activities. In the APPS work stream, each African partnership hospital conducts a patient safety situational analysis, which includes an understanding of the contextualized evidence-policy interface in each country [34]. Partnerships then focus on specific patient safety activities, keeping a clear view on how these activities might further strengthen the evidence-policy interface. Evidence-based patient safety interventions in partnership hospitals strengthen the case for evidence-based policies at a national level, hence elevating partnership hospitals as "amplification points" for spread. Each of the six first wave APPS hospital partnerships has utilized situational analysis findings as an entry point to engage with decision-makers. For example, in Ethiopia, patient safety situational analysis findings from Gondar Hospital were harnessed to influence development of patient safety policy and national adoption of the patient safety improvement approach. This highlighted the strong and direct influence front-line practitioners have in the policy arena.

Second, conduct rapid stakeholder analyses. This involves mapping and analyzing evidence-policy stakeholders, as well as the relationships between them. In the APPS experience, the aim has been to develop a strategic approach to engaging patient safety stakeholders. APPS partnerships focus on engaging relevant stakeholders through the utilization of real implementation experience in African hospitals. Early experiences show the importance of mapping out key entry points to influence and strengthen evidence-policy linkages in patient safety. Stakeholder engagement has focused on both national and sub-national decision makers, recognizing that patient safety policy-making is complex and includes both formal and informal decision making processes. This is particularly significant where decision-making power resides in networks of “important people.”

Third, synthesize other evidence-policy experiences. The programme carries this out by examining literature and discussing with key informants, as well as by prioritizing close linkages with academic institutions interested in strengthening the evidence-policy interface. APPS partnership hospitals build on the work of knowledge brokers within prominent institutions, such as ministries of health, to capitalize on the significant agency capacity and potential found in such institutions. Close working relationships with various African ministries of health has allowed for knowledge flow on patient safety improvement and enhanced alignment of existing national patient safety endeavours. These avenues generate ripples that are critical in influencing change outside the APPS partnership hospital. In fact, experiences from the first wave APPS partnerships have been utilized in developing a tool for patient safety policy making across the African Region, a unique example of how front line implementation can influence national and regional policy directions.

Fourth, utilize evidence-policy platforms. Existing evidence-policy platforms can enhance trust and communication between evidence generators and policy makers. In the patient safety context, these platforms have been crucial in integrating health service delivery within the context of other health systems issues, thus generating a culture of joint accountability between scientists and health policy makers. In this respect, country collaboration between APPS partnership hospitals and national knowledge brokers is a clear mechanism for synergy. For example, early collaboration between APPS and various national platforms resulted in patient safety policy dialogue in each of the six African countries. Building on these experiences, coordinated efforts between APPS and WHO EVIPNet have resulted in evidence-policy capacity workshops, such as the Annual Meeting of the International Society for Quality in Health Care in 2012.

Fifth, focus on the use of "evidence-policy" tools. Availability of a large body of well synthesized, easy-to-understand evidence on patient safety issues adds to the "transferability" of that evidence. Knowledge translation tools can help transform evidence for easier use by knowledge brokers seeking to address specific patient safety issues. These tools can also serve as a common platform for action within participating countries, as has been the case for hand hygiene improvement throughout the world [35,36]. Along these lines, the APPS Resource Map, framed around the 12 patient safety action areas, provides a well-understood frame of reference for both policy makers and implementers [37]. Key evidence-policy tools are freely available for use through this APPS Resource Map.

Sixth, aim to feed the knowledge pool. This highlights the need to generate and share evidence on patient safety, in alignment with national health priorities. Evaluation mechanisms can help measure results and provide feedback on effectiveness of evidence-policy strengthening practices. Sharing experiences through various knowledge dissemination methods, including seminars and publications, facilitate spread of action. Each of the first wave APPS partnerships has utilized change experiences to feed the local and global pools of knowledge. Interestingly, the learning has started to capture the attention of those interested in how change mechanisms in the developing world can inform thinking in the so-called developed world – a phrase commonly being referred to as 'reverse innovation in global health systems’.

Finally, aim to generate a “ripple effect”. Powerful advocacy networks can bring evidence to the fore of policy-making agendas. Close in-country linkages with a variety of stakeholders, including the media, are indispensible when disseminating learning on how to strengthen the interface between evidence and policy. Alignment with national policies is at the core of such a ripple. APPS partnership experiences have successfully captured the attention of national and global audiences. The 2012 World Health Assembly marked the 10^th^ anniversary of the global resolution on patient safety. Sir Liam Donaldson, the World Health Organization’s Envoy for Patient Safety addressed the Assembly in an illustrious gathering. His keynote was succeeded by a speech from the Medical Superintendent of Kisiizi Hospital in rural Uganda that is partnered with Countess of Chester Hospital, England. Patient safety experiences from the Kisiizi-Chester partnership are a clear testimony to the common problems and solutions in global patient safety – and serve to illustrate the clear ripple effect between the local and global policy making arenas.

### A new model for partnerships?

Employing two-way interactive practices where the production of evidence and information is based on bidirectional relationships and learning has allowed APPS partnership hospitals to play critical roles as knowledge brokers and "amplification points"—both in their local settings and beyond. This work is built on the premise that improved ways of organizing and sharing evidence can help achieve the translation of multi-faceted knowledge into policy. The APPS model is unique in employing partnerships to promote the two-way sharing of tools, processes and learning as a means of improving patient safety in African hospitals. The APPS commitment to strengthening the evidence-policy interface rests upon sustainably and successfully influencing patient safety policy-making processes. Each of the six APPS partnerships locally contextualizes their work and develops partnership plans. In our experience, this has been a key enabler in early efforts to catalyze policy change in patient safety.

The programme has of course faced significant challenges that cannot be overlooked. Three difficulties stand out in particular that focus on the areas of resources and capacity, stakeholder shifts, and policy alignment. First, given that the capacity of an African partnership hospital is limited, the foremost priority of the hospital patient safety team is to tackle the real and immediate challenges faced rather than focus on being a national patient safety change agent. Second, due to the nature of policy-making and political cycles, rapid shifts in evidence-policy stakeholders are common, which makes policy dialogue and strategic engagement by the partnership difficult. Third, patient safety is a relatively new arena and hospital partnerships are often considered as being peripheral endeavours that operate outside of national health agendas. Aligning and integrating health policies requires continued dialogue to overcome closed mindsets—in spite of limited time and resources.

Despite these challenges, early experiences with the APPS model of hospital-to-hospital partnerships can help inform other programmes on how to incorporate evidence-policy considerations in programme planning. In particular, focused planning on evidence-policy strengthening during the design phase of programmes aiming to improve health service delivery can allow “partnership improvement experiences” to feed into the evidence-policy interface. This highlights the importance of “bottom up” strengthening of the linkage between evidence and policy, particularly in the African Region. Further, evidence-policy strengthening is an area ripe for bidirectional learning within and between partnership, in keeping with the principles and spirit of global health today. Indeed, as has been the case for APPS, lessons and evidence from rapidly evolving health systems in Africa can help inform policy-making across the world.

## Competing interests

The authors declare that they have no competing interests.

## Authors’ contribution

SBS drafted the original manuscript. VD provided extensive revisions. All authors provided content to manuscript development. SBS, JS, JDH, RG and EK were part of a team that conceptualized the approach described in the manuscript. All authors read and approved the final manuscript.

## References

[B1] Koplan JP Bond TC Merson MH Reddy KS Rodriguez MH Sewankambo NK Wasserheit JN Towards a common definition of global health Lancet 2009 373 1993 5 10.1016/S0140-6736(09)60332-9 19493564 PMC9905260

[B2] Crisp N Turning the world upside down: the search for global health in the twenty-first century 2010 London: Royal Society of Medicine Press

[B3] Immelt J Govindarajan V Trimble C How GE is disrupting itself Harvard Bus Rev 2009 87 56 65

[B4] Marcus A To Fix Health Care, Some Study Developing World 2009 [http://online.wsj.com/article/SB124648865046182847.html]

[B5] Berwick DM Lessons from developing nations on improving health care BMJ 2004 328 1124 1129 10.1136/bmj.328.7448.1124 15130984 PMC406330

[B6] Syed D Dadwal V Rutter P Storr J Hightower J Gooden R Carlet J Nejad S Kelley E Donaldson L Pittet D Developed-developing country partnerships: benefits to developed countries? Globalization and Health 2012 8 17 10.1186/1744-8603-8-17 22709651 PMC3459713

[B7] Choi BC Pang T Lin V Puska P Sherman G Goddard M Ackland MJ Sainsbury P Stachenko S Morrison H Can scientists and policy makers work together? J Epidemiol Community Health 2005 59 632 637 10.1136/jech.2004.031765 16020638 PMC1733111

[B8] Brownson RC Royer C Ewing R McBride TD Researchers and policymakers: travellers in parallel universes Am J Prev Med 2006 30 2 164 72 10.1016/j.amepre.2005.10.004 16459216

[B9] Haines A Kuruvilla S Borchert M Bridging the implementation gap between knowledge and action for health Bull World Health Organ 2004 82 724 31 15643791 PMC2623035

[B10] Nuyens Y Lansang MA Knowledge translation: linking the past to the future Bull World Health Organ 2006 84 590 10.2471/BLT.06.033969 16917638 PMC2627426

[B11] Koon A Nambiar D Ra K Embedding of research into decision-making processes 2012 New Delhi: Public Health Foundation of India

[B12] Colon-Ramos U Lindsay AC Monge-Rojas R Greaney ML Campos H Peterson KE Translating research into action: a case study on trans fatty acid research and nutrition policy in Costa Rica Health Policy Plan 2007 22 363 374 10.1093/heapol/czm030 17951318

[B13] Lavis JN Research, public policymaking, and knowledge-translation processes: Canadian efforts to build bridges J Contin Educ Health Prof 2006 26 37 45 10.1002/chp.49 16557509

[B14] Aaserud M Lewin S Innvaer S Paulsen E Dahlgren AT Trommald M Duley L Zwarenstein M Oxman AD Translating research into policy and practice in developing countries: a case study of magnesium sulfate for preeclampsia BMC Health Serv Res 2005 5 68 10.1186/1472-6963-5-68 16262902 PMC1298297

[B15] Lavis JN Lomas J Hamid M Sewankambo NK Assessing country-level efforts to link research to action Bull. World Health Organ 2006 84 620 628 10.2471/BLT.06.030312 16917649 PMC2627430

[B16] Hyder AA Bloom G Leach M Syed SB Peters DH Exploring health systems research and its influence on policy processes in low income countries. BMC Public Health 2007 7 309 10.1186/1471-2458-7-309 17974000 PMC2213669

[B17] Varvasovszky Z Brugha R A stakeholder analysis Health Policy Plan 2000 15 338 45 10.1093/heapol/15.3.338 11012410

[B18] Dobbins M Robeson P Ciliska D Hanna S Cameron R O'Mara L DeCorby K Mercer S A description of a knowledge broker role implemented as part of a randomized controlled trial evaluating three knowledge translation strategies Implementation Science 2009 4 23 10.1186/1748-5908-4-23 19397820 PMC2680804

[B19] World Health Organization Evidence-informed policy-making 2013 What is evidence-informed policy-making? http://www.who.int/evidence/about/en/index.html

[B20] Hamid M Bustamante-Manaog T Truong VD Akkhavong K Fu H Ma Y Zhong X Salmela R Panisset U Pang T EVIPNet: translating the spirit of Mexico Lancet 2005 366 1758 60 10.1016/S0140-6736(05)67709-4 16298204

[B21] Evidence Informed Policy Network. (2013) EVIPNet 2013 from Resources for evidence-informed policy http://www.evipnet.org

[B22] Dixon-Woods M Why is patient safety so hard? A selective review of ethnographic studies J Health Serv Res Policy 2010 15 11 16 10.1258/jhsrp.2009.009041 20075122

[B23] Benning A Ghaleb M Suokas A Dixon-Woods M Dawson J Barber N Franklin BD Girling A Hemming K Carmalt M Rudge G Naicker T Nwulu U Choudhury S Lilford R Large scale organisational intervention to improve patient safety in four UK hospitals: mixed method evaluation BMJ 2011 3 342 10.1136/bmj.d195 PMC3033440 21292719

[B24] Titler M The Evidence for Evidence-Based Practice Implementation. In R. Hughes, Patient Safety and Quality: An Evidence-Based Handbook for Nurses (Chapter 7) 2008 Rockville, MD: U.S. Agency for Healthcare Research and Quality

[B25] Davidoff F Heterogeneity is not always noise: lessons from improvement JAMA 2009 302 2580 10.1001/jama.2009.1845 20009058

[B26] Jha AK Prasopa-Plaizier N Larizgoitia I Bates DW Research Priority Setting Working Group of the WHO World Alliance for Patient Safety. **Patient safety research**: **an overview of the global evidence** Qual Saf Health Care 2010 19 1 42 7 10.1136/qshc.2008.029165 20172882

[B27] WHO African Region Patient Safety in African Health Services: Issues and Solutions, Report of the Regional Director to the 58th WHO AFRO Regional Committee 2008 Brazzaville: WHO Regional Office for Africa

[B28] Entea C Oyewumiband A Mpora OB Healthcare professionals' understanding and awareness of patient safety and quality of care in Africa: a survey study J Risk Saf Med 2010 22 103 110

[B29] Grimshaw JM Shirran L Thomas R Mowatt G Fraser C Bero L Grilli R Harvey E Oxman A O'Brien MA Changing provider behavior: an overview of systematic reviews of interventions Medical Care 2001 39 8 Suppl 45 11583120

[B30] Syed SB Gooden R Storr J Hightower JD Rutter P Bagheri Nejad S Lardner A Kelley E Pittet D African Partnerships for Patient Safety: a vehicle for enhancing patient safety across two continents World Hosp Health Serv 2009 45 1 24 27 20411829

[B31] World Health Organization African Partnerships for Patient Safety 2013 http://www.who.int/patientsafety/implementation/apps/en/index.html

[B32] Armstrong K Kendall E Translating knowledge into practice and policy: the role of knowledge networks in primary health care Health Inf Manage J 2010 39 2 9 17 10.1177/183335831003900203 20577019

[B33] World Health Organization Strengthening the evidence-policy interface: the APPS approach from African Partnerships for Patient Safety 2013 http://www.who.int/patientsafety/implementation/apps/resources/APPS_approach_evidence-policy_full_2010-12_EN.pdf

[B34] World Health Organization Patient Safety Situational Analysis. African Partnerships for Patient Safety 2013 http://www.who.int/patientsafety/implementation/apps/resources/APPS_Improv_PS_Situational_Analysis_LF_2012_04_EN.pdf

[B35] Pittet D Donaldson L Clean care is safer care: a worldwide priority Lancet 2005 366 1246 7 10.1016/S0140-6736(05)67506-X 16214584 PMC7134620

[B36] World Health Organization WHO Guidelines on hand hygiene in health care 2009 Geneva: World Health Organization

[B37] World Health Organization Patient safety resource map. African Partnerships for Patient Safety 2013 http://www.who.int/entity/patientsafety/implementation/apps/resources/APPS_resource_map.pdf

